# RNA-Sequencing Analysis of TCDD-Induced Responses in Zebrafish Liver Reveals High Relatedness to *In Vivo* Mammalian Models and Conserved Biological Pathways

**DOI:** 10.1371/journal.pone.0077292

**Published:** 2013-10-30

**Authors:** Zhi-Hua Li, Hongyan Xu, Weiling Zheng, Siew Hong Lam, Zhiyuan Gong

**Affiliations:** 1 Department of Biological Sciences, National University of Singapore, Singapore; 2 Yangtze River Fisheries Research Institute, Chinese Academy of Fishery Sciences, Wuhan, China; Auburn University, United States of America

## Abstract

TCDD is one of the most persistent environmental toxicants in biological systems and its effect through aryl hydrocarbon receptor (AhR) has been well characterized. However, the information on TCDD-induced toxicity in other molecular pathways is rather limited. To fully understand molecular toxicity of TCDD in an in vivo animal model, adult zebrafish were exposed to TCDD at 10 nM for 96 h and the livers were sampled for RNA-sequencing based transcriptomic profiling. A total of 1,058 differently expressed genes were identified based on fold-change>2 and TPM (transcripts per million) >10. Among the top 20 up-regulated genes, 10 novel responsive genes were identified and verified by RT-qPCR analysis on independent samples. Transcriptomic analysis indicated several deregulated pathways associated with cell cycle, endocrine disruptors, signal transduction and immune systems. Comparative analyses of TCDD-induced transcriptomic changes between fish and mammalian models revealed that proteomic pathway is consistently up-regulated while calcium signaling pathway and several immune-related pathways are generally down-regulated. Finally, our study also suggested that zebrafish model showed greater similarity to *in vivo* mammalian models than *in vitro* models. Our study indicated that the zebrafish is a valuable in vivo model in toxicogenomic analyses for understanding molecular toxicity of environmental toxicants relevant to human health. The expression profiles associated with TCDD could be useful for monitoring environmental dioxin and dioxin-like contamination.

## Introduction

Dioxin-like compounds are major environmental contaminants that could pose serious threats to public health and the ecosystem [1]. Since 1980s, it has been increasingly documented that dioxin-like compounds cause various biological effects in laboratory animals and human [2], [3]. Among them, 2,3,7,8-tetrachlorodibenzo-*p*-dioxin (TCDD) is the most potent toxicant and it is produced from both natural and anthropogenic processes including incineration of chlorine-containing substances, bleaching of paper, manufacturing of specific organochlorine chemicals, volcanoes, and forest fires [4]. As an aromatic hydrocarbon, TCDD has a long biological half-life and is heavily accumulated in the food chain, which causes adverse effects on human health at environmental levels [4], [5]. Till now, many animal models, including fishes, have been used in TCDD studies [6], [7]. However, most of these studies have focused on the physiological-biochemical parameters and typical molecular markers, such as the gene *cyp1a* and the Aryl hydrocarbon receptor (AhR) signaling pathway [7] and detailed molecular toxicity of TCDD remains to be elucidated.

Genomic approaches have been increasingly used in toxicological research in the past decade. Previous toxicogenomic studies mostly used DNA microarray technology to capture the global gene expression data and to evaluate the effects of toxicant exposure [8], [9]. However, there are several drawbacks in microarray analysis, e.g. limited sensitivity, probe cross-hybridization, incomplete genome coverage and a prerequisite for sequence information in order to include new probes [10]. Recently, the advent of next-generation sequencing (NGS) technologies has significantly accelerated genomic research and provided a better alternative for transcriptomic analysis [11]. By high-throughput RNA sequencing, it is feasible to measure transcript abundance and transcriptomic profiles with a broad dynamic range, therefore providing a powerful tool to determine the potential adverse effects of environmental contaminants on public health [8].

Comparative studies across different taxonomic groups are important not only in understanding of organism diversity but also for inferring important biological responses due to evolutionary conservation [4]. In toxicology, animal models are widely used to infer human responses to chemical exposure. Particularly, it is valuable to use multiple animal models for comparative analyses in order to determine conserved adverse responses or molecular events. As lower vertebrates in evolution, fishes are particularly important models for such comparative studies [12], [13]. Now the zebrafish (*Danio rerio*) has become an increasingly popular model in human disease studies because of its many advantageous properties in laboratory experiments, such as its small size and easy availability in a large number, short generation time for genetic manipulation, and cost effectiveness for high-throughput studies. More importantly, many molecular and developmental studies have shown that zebrafish and human share many common genes in conserved developmental pathways in organogenesis and related physiological processes as well as in carcinogenesis [14], [15].

As there is so far no NGS based transcriptomic analysis for molecular response to TCDD treatment, in the present study we carried out RNA sequencing analyses of TCDD-treated in order to carry out a genome-wide identification of novel TCDD responsive genes and pathways, which should provide a comprehensive understanding of the molecular mechanism of TCDD-induced toxicity in mammals and human. We further compared the zebrafish response with those from mammalian systems and thus identified common molecular pathways deregulated in both fish and mammals. We found that the zebrafish model is more similar to mammalian in vivo models than in vitro models, thus indicating a validity of the zebrafish as an emerging in vivo model in comparative toxicological research.

## Materials and Methods

### Zebrafish

Experimental procedures were carried out following the approved protocol by Institutional Animal Care and Use Committee of National University of Singapore (Protocol 079/07). Adult zebrafish (3-month old), which are Singapore wild type zebrafish, were purchased from a local aquarium farm (Mainland Tropical Fish Farm, Singapore) and acclimated for at least one week in our aquarium before chemical exposure experiment. Fish were maintained at ambient temperature of around 28°C with a 14-h light and 10-h dark cycle in a flow-through water system.

### Chemical exposure

TCDD was purchased from Sigma-Aldrich (USA) and dissolved in dimethyl sulfoxide (DMSO). Male adult fish were used in the exposure experiments by immersing in the TCDD water (10 nM) for 96 h at ambient temperature (28°C) in a static condition. Control fish were kept in water with 0.01% DMSO (vehicle) under the same condition. Water was changed daily throughout the treatment. After 96 hours of chemical exposure, treated and control fish were sacrificed and liver samples were dissected from each fish.

### RNA sample preparation and SAGE library sequencing

Total RNA was extracted from livers (excluding gall bladders) of individual fish using TRIzol® Reagent (Invitrogen) and treated with DNase I (Invitrogen) to remove genomic DNA contamination. For RNA sequencing, RNA was pooled equally from 9 fish for each group (TCDD and control). Poly A+ RNA was purified using Dynabeads® Oligo (dT) EcoP (Invitrogen) and subjected to cDNA synthesis. Synthesized cDNA was digested by NlaIII and sequencing adapters were added to the cDNA fragments. SAGE (serial analysis of gene expression) sequencing (tag length = 27 nucleotides) was performed by Mission Biotech (Taiwan) with ABI SOLiD^TM^ System 2.0 (Applied Biosystems). The RNA-sep data reported in the present study was submitted to Gene Expression Omnibu with an access number GSE49915.

### Gene annotation and selection of differentially expressed genes

All SAGE tags were mapped to the zebrafish Reference Sequence database (http://www.ncbi.nlm.nih.gov/RefSeq) with maximum 2 nucleotide mismatch. Uniquely mapped tag counts for each transcript were normalized to TPM (transcripts/tags per million). For biological implication analyses, genes with only marginal expression, as defined by TPM<10 in both control and TCDD groups, were excluded. As it has been previously reported that the actual measured quantity of differential expression (fold change or ratio) is more consistent and reproducible in identifying differentially expressed genes than the statistical significance (p-value) [16], [17], in the present study, we selected differentially expressed genes based on fold change>2 and TPM>10.

### Gene Ontology enrichment analysis

Gene ontology enrichment analysis was performed using DAVID (The Database for Annotation, Visualization and Integrated Discovery) with the total zebrafish genome information as the background and p-values based on a modified Fisher's exact t-test. Gene Ontology Fat categories were used for this analysis and the cut-off p-value is 0.05.

### Real-time PCR

Real-time PCR was performed using the LightCycler system (Roche Applied Science) with LightCycler FastStart DNA Master SYBR Green I (Roche Applied Science) according to the manufacturer's instruction. For comparison between real-time PCR and RNA-seq results, Cp (crossing point value) and TPM values for each gene were normalized against Cp and TPM of *β-actin1* (GI_ID:18858334).

### Analysis of the hepatic enriched gene list by GSEA and IPA analysis

GSEA (Gene Set Enrichment Analysis) pre-ranked option was used to analyze the entire set of differentially expressed genes (651 up- and 407 down-regulated genes). Briefly, the gene symbols of human homologs of the enriched zebrafish Unigene clusters were ranked using logarithm transformed fold change (base 2). The number of permutation used was 1000. Pathways with false discovery rate (FDR) <0.25 were considered statistically significant. For IPA (Ingenuity Pathway Analysis), the same set of differentially expressed genes was uploaded to online Ingenuity Pathways Knowledge Base for functional implication analyses.

### Cross-species comparison

Six sets of transcriptomic data for *in vivo* and *in vitro* mammalian models treated by TCDD (GSE10082, GSE10083, GSE10769, GSE10770, GSE14555 and GSE34251) were retrieved from GEO (Gene Expression Omnibus). GSEA was used to establish the relatedness between zebrafish and mammalian models. The zebrafish hepatic transcriptome lists were converted into human and mouse homolog Unigene clusters. The statistical significance of the enrichment score was estimated by using an empirical phenotype-based permutation test. An FDR value was provided by introducing adjustment of multiple hypothesis testing.

## Results and Discussion

### General features of SAGE tags in TCDD-treated and control groups

**Figure 1:**
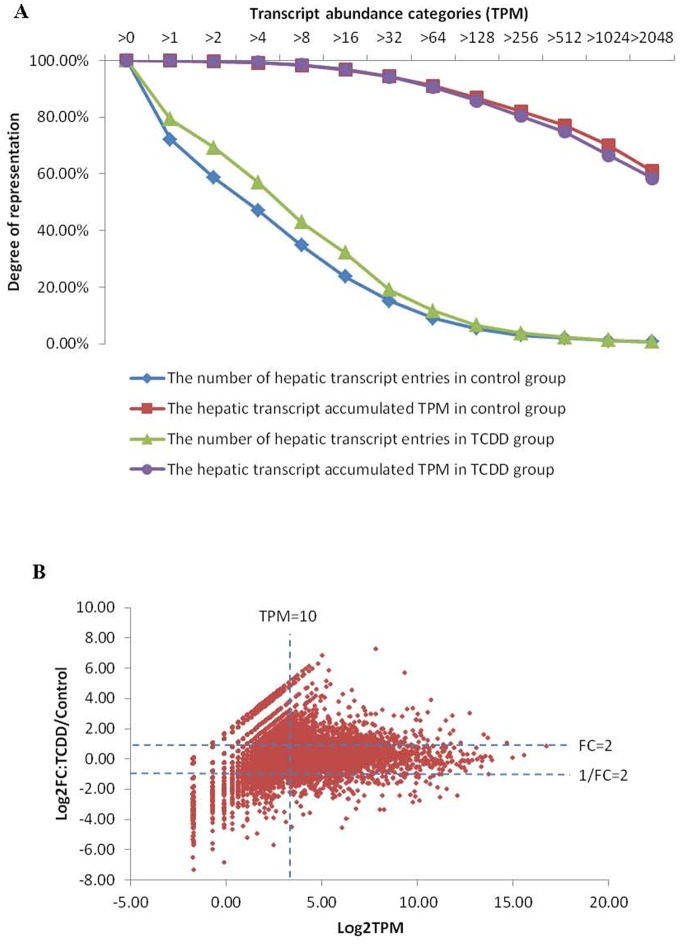
Comparison of transcriptomic profiles between TCDD and control groups. (A) Distribution of transcript entries and total transcript counts over different tag abundance categories in liver of zebrafish. The percentages of total transcript counts and number of different transcript entries per category are plotted on a log scale (base10). (B) Relationship between the hepatic transcriptome changing range and its expression level in zebrafish after TCDD treatment. The base of log value is 2.

There were 11.7 million and 17.9 million SAGE sequence tags generated from the DMSO vehicle control and TCDD-treated groups, respectively. About 2.7 million tags in the control group and 3.2 million tags in the TCDD group could be mapped with the known transcripts. The mapped tags were normalized to transcripts per million (TPM) and the expression level of genes ranged from 0.3 to 110844.2 TPM in the two groups, indicating a dynamic range of more than six orders of magnitude in transcript abundance. As shown in Figure 1A, transcript abundance profiles in both groups were very similar. The transcriptomes consist of a small number of high abundant transcripts and a large number of low abundant transcripts, similar to those reported in many previous RNA-seq studies [18], [19]. The percentages of the transcripts with relatively high expression level (TPM>256) were 3.8% in the TCDD group and 3.1% in the control group, but contributed to 80.4% and 83.0% of the total transcripts respectively. In contrast, the percentages of the transcripts with the low expression level (TPM<16) were 67.1% and 76.3% in the TCDD group and control group, contributing only 3.1% and 3.2% of the total transcripts.

### Differentially expressed genes in response to TCDD and their gene ontology profile

**Figure 2:**
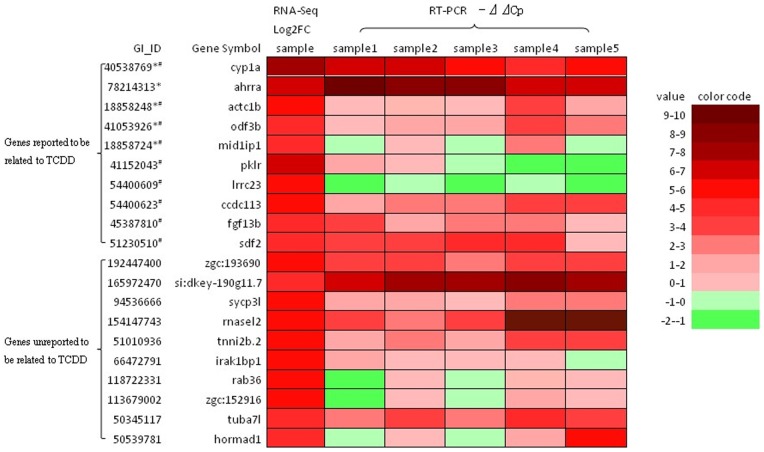
RT-qPCR validation of top 20 up-regulated TCDD-induced genes. RT-qPCR was performed from five individual liver samples collected from five fish treated by TCDD from a new experiment. The relative expression level of the genes was shown in color code as indicated on the fight and the value is in log2 fold change as compared with a housekeeping gene, *β-actin1*. *-only reported in fish model; #-only reported in mammalian model.

Figure 1B shows the relationship between the dynamic transcript fold-change of hepatic transcriptome and its expression level in zebrafish treated by TCDD. Differentially expressed genes after TCDD treatment were first determined by comparison of the two sets of mapped SAGE tags with fold-change>2 and TPM >10. In total, 1,058 genes were identified, including 651 up-regulated genes and 407 down-regulated genes. The top 20 most up-regulated transcripts based on fold-change are listed in the Table 1. After compared with TCDD responsive genes in CTD (The Comparative Toxicogenomics Database, http://ctdbase.org/), ten gens (*cyp1a, ahrra, actc1b, odf3b, mid1ip1, pklr, lrrc23, ccdc113, sdf2, and fgf13*) have been reported to be related in fish and/or mammalian models after TCDD exposure among the top 20 genes. Not surprisingly, *cyp1a*, the best known molecular marker for TCDD exposure [20], was the most up-regulated gene (151.4 fold) in the list, indicating that our TCDD treatment in the experiment was effective. It is interesting to note that *adrra* (aryl hydrocarbon receptor repressor a) is the second highest up-regulated gene, while the two aryl hydrocarbon receptor genes (*ahr1a* and *ahr2*) were not significantly up-regulated in our RNA-seq data with a modest 50% increase for *adr2* and slightly decrease for *adr1a* (data not shown), 10 novel TCDD-responsive genes, eight annotated (*rab36, sycp3l, rnasel2, tnni2b.2, hormad1, tuba7l, irak1bp1* and zgc:152916,) and two unannotated (zgc:193690 and si:dkey-190g11.7), have been identified from the top 20 most up-regulated genes. To confirm their inducibility of the 20 genes by TCDD, real-time PCR was carried out with individual fish liver samples from an independent set of TCDD treatment experiment. As shown in Figure 2, 17 out of 20 genes showed up-regulation in at least three individual samples; among them, 13 genes had up-regulation in all of the five individual samples. Thus, our RT-qPCR data indicated a strong agreement with the RNA-sequencing data and, more importantly, the validation was from five independent biological samples. Interestingly, all of the 10 novel TCDD responsive genes found in the present study were validated by RT-qPCR (Figure 2). Thus, our RNA-seq data provided additional biomarker genes for TCDD exposure. Among these validated biomarker genes, several of them apparently encodes secreted protein (e.g. *fgf13b* and *rnasel2*) and their protein products are also likely present in the circulating blood and may offer convenient non-invasive assays for detection of TCDD exposure. A notable exception in the validation experimental data was *lrrc23* that was constantly down-regulated in all five individuals, indicating that *irrc23* is not a reproducibly up-regulated gene by TCDD. In future, these novel molecular markers for TCDD exposure can be further tested for their time- and dose-responsiveness.

**Table 1:** Top up-regulated genes by TCDD in zebrafish liver.

GI_ID	Gene Symbol	Gene Name	TPM Value		Log2FC
			Control	TCDD	
40538769	cyp1a	cytochrome P450, family 1, subfamily A	1.49	225.60	7.24
41152043	pklr	pyruvate kinase, liver and RBC	0.37	28.99	6.28
78214313	ahrra	aryl-hydrocarbon receptor repressor a	0.30	20.48	6.09
118722331	rab36	RAB36, member RAS oncogene family	0.37	23.32	5.97
94536666	sycp3l	synaptonemal complex protein 3 like	0.30	18.27	5.93
113679002	zgc:152916	zgc:152916	0.37	20.80	5.80
154147743	rnasel2	ribonuclease like 2	12.65	641.82	5.67
192447400	zgc:193690	zgc:193690	0.30	13.55	5.50
54400609	lrrc23	leucine rich repeat containing 23	0.30	12.29	5.36
66472791	irak1bp1	interleukin-1 receptor-associated kinase 1 binding protein 1	0.30	11.97	5.32
51010936	tnni2b.2	troponin I, skeletal, fast 2b.2	0.30	11.34	5.24
54400623	ccdc113	coiled-coil domain containing 113	0.37	13.86	5.22
18858248	actc1b	actin, alpha, cardiac muscle 1b	1.12	39.07	5.13
41053926	odf3b	outer dense fiber of sperm tails 3B	0.37	11.66	4.97
165972470	si:dkey-190 g11.7	si:dkey-190g11.7	0.74	23.00	4.95
51230510	sdf2	stromal cell-derived factor 2	1.49	45.06	4.92
45387810	fgf13b	fibroblast growth factor 13b	0.37	10.40	4.80
50539781	hormad1	HORMA domain containing 1	1.49	39.39	4.73
50345117	tuba7l	tubulin, alpha 7 like	0.74	19.53	4.71
18858724	mid1ip1	MID1 interacting protein 1	0.74	18.27	4.62

Besides *cyp1a*, some other genes with high abundance were also up-regulated by TCDD exposure in zebrafish (Table S1). The up-regulation of *pklr* (pyruvate kinase, liver and red blood cell) in our experiment was consistent with several previous reports that TCDD affects pyruvate utilization for energy and thus glycolysis and gluconeogenesis [21], [22]. Gene *e*xpression of *alas1*, the first and rate-limiting enzyme involved in heme biosynthesis [23], was up-regulated in our study, suggesting that the heme biosynthesis was deregulated in hepatic cells of fish exposed to TCDD. Hspa5 is a member of the heat shock protein 70 (Hsp70) family and it has been used as an endoplasmic reticulum (ER) stress sensor [24]. In our study, the expression of *hspa5* was up-regulated with 10.2 fold, implying TCDD led to the ER stress and this event was a highly conserved adaptive response induced by environmental toxicants. Moreover, the gene *pck1* (phosphoenolpyruvate carboxykinase 1), as a key factor in gluconeogenesis, was up-regulated 6.8 fold, indicating that TCDD exposure also affected the gluconeogenesis by altering the expression of genes encoding key gluconeogenic enzymes [25].

Through gene ontology analysis, the differentially expressed genes were classified into different categories based on GO database using DAVID software (Table 2). Among the up-regulated GO categories, the most significant ones included Endoplasmic reticulum (p-value = 1.19E-13) and Proteasome complex (p-value = 2.67E-09). Since most P450s (e.g. CYP1A enzymes) are involved in xenobiotic metabolism and primarily located in endoplasmic reticulum, it is not surprising that the TCDD-induced physiological stress occurred mainly in these cellular components, which was also supported by the up-regulated 19 genes of CYP family in our study (Table S2). Moreover, other histological studies also found TCDD led to hypertrophy of hepatocytes, glycogen depletion and lipidosis of liver in zebrafish and other fish [26], which were related to the endoplasmic reticulum (ER) stress via some cellular receptor or/and protein [27], [28]. In subsequent analysis, our results also showed that the proteasome related pathways (Proteasome pathway and HSA03050 Proteasome, see Table 3) were significantly up-regulated in zebrafish after TCDD treatment. Consistent with this, several GO categories involved in Proteasome deregulation, such as Protein folding and Cellular protein catabolic process in the Biological Process category, as well as Unfolded protein binding in the Molecular Function category, further indicating that the proteasome related bio-functions were deregulated.

**Table 2:** Enriched GO terms in response to TCDD treatment in zebrafish liver (p<0.01).

Category	Term	Count	%	P-Value	Fold Enrichment
**Biological**	protein folding	19	2.98	1.05E-06	3.94
**process**	cellular macromolecule catabolic process	24	3.77	4.61E-05	2.59
	macromolecule catabolic process	26	4.08	5.61E-05	2.44
	proteolysis involved in cellular protein catabolic process	22	3.45	7.17E-05	2.65
	cellular protein catabolic process	22	3.45	7.17E-05	2.65
	modification-dependent protein catabolic process	20	3.14	1.65E-04	2.66
	modification-dependent macromolecule catabolic process	20	3.14	1.65E-04	2.66
	protein catabolic process	22	3.45	2.53E-04	2.43
	cell cycle	19	2.98	4.11E-04	2.56
	protein maturation by peptide bond cleavage	4	0.63	8.39E-04	17.76
	proteolysis	41	6.44	1.40E-03	1.66
	protein transport	26	4.08	1.59E-03	1.96
	establishment of protein localization	26	4.08	1.59E-03	1.96
	protein localization	27	4.24	1.80E-03	1.91
	response to bacterium	8	1.26	2.08E-03	4.33
	intracellular transport	19	2.98	2.14E-03	2.22
	cellular protein localization	15	2.35	4.82E-03	2.33
	cellular macromolecule localization	15	2.35	5.13E-03	2.31
**Cellular**	endoplasmic reticulum	42	6.59	1.19E-13	3.67
**component**	proteasome complex	16	2.51	2.67E-09	6.93
	cytosol	21	3.30	6.39E-07	3.65
	endoplasmic reticulum part	14	2.20	6.83E-06	4.57
	endoplasmic reticulum membrane	11	1.73	1.89E-04	4.25
	nuclear envelope-endoplasmic reticulum network	11	1.73	3.44E-04	3.96
	proteasome core complex	6	0.94	4.41E-03	5.31
**Molecular**	unfolded protein binding	12	1.88	2.85E-05	4.83
**function**	oligosaccharyl transferase activity	4	0.63	5.94E-04	19.98
	acid-amino acid ligase activity	12	1.88	1.74E-03	3.06
	threonine-type peptidase activity	6	0.94	2.23E-03	6.24
	threonine-type endopeptidase activity	6	0.94	2.23E-03	6.24
	dolichyl-diphosphooligosaccharide-protein lycotransferase activity	3	0.47	4.64E-03	24.98
	ligase activity, forming carbon-nitrogen bonds	13	2.04	5.65E-03	2.50
	translation initiation factor activity	8	1.26	5.92E-03	3.63
	heme binding	12	1.88	8.31E-03	2.50
	RNA binding	20	3.14	9.61E-03	1.89

**Table 3:** Significantly regulated pathways in zebrafish after TCDD treatment, by GSEA analysis with cutoff FDR<0.25.

Pathways	NES	NOM p-value	FDR q-value
G1 to S cell cycle reactome	1.94	<0.001	0.024
HSA04110 cell cycle	1.9	<0.001	0.025
Cell cycle KEGG	1.86	0.003	0.031
Proteasome pathway	1.81	0.007	0.053
HSA03050 Proteasome	1.74	0.007	0.115
HSA03010 Ribosome	−2.31	<0.001	<0.001
Ribosomal proteins	−2.21	<0.001	0.001
HSA04020 Calcium signaling pathway	−1.85	0.005	0.126
Prostaglandin synthesis regulation	−1.81	0.005	0.167
ST FAS signaling pathway	−1.81	0.007	0.139
ST T cell signal transduction	−1.79	<0.001	0.135
HSA04650 Natural killer cell mediated cytotoxicity	−1.76	0.007	0.160
HSA04660 T cell receptor signaling pathway	−1.75	0.008	0.152

Note: NES, normalized enrichment scores; NOM p-value, nominal p-value for enrichment; FDR, false discovery rate.

### Change of transcription factor networks by TCDD exposure

**Figure 3:**
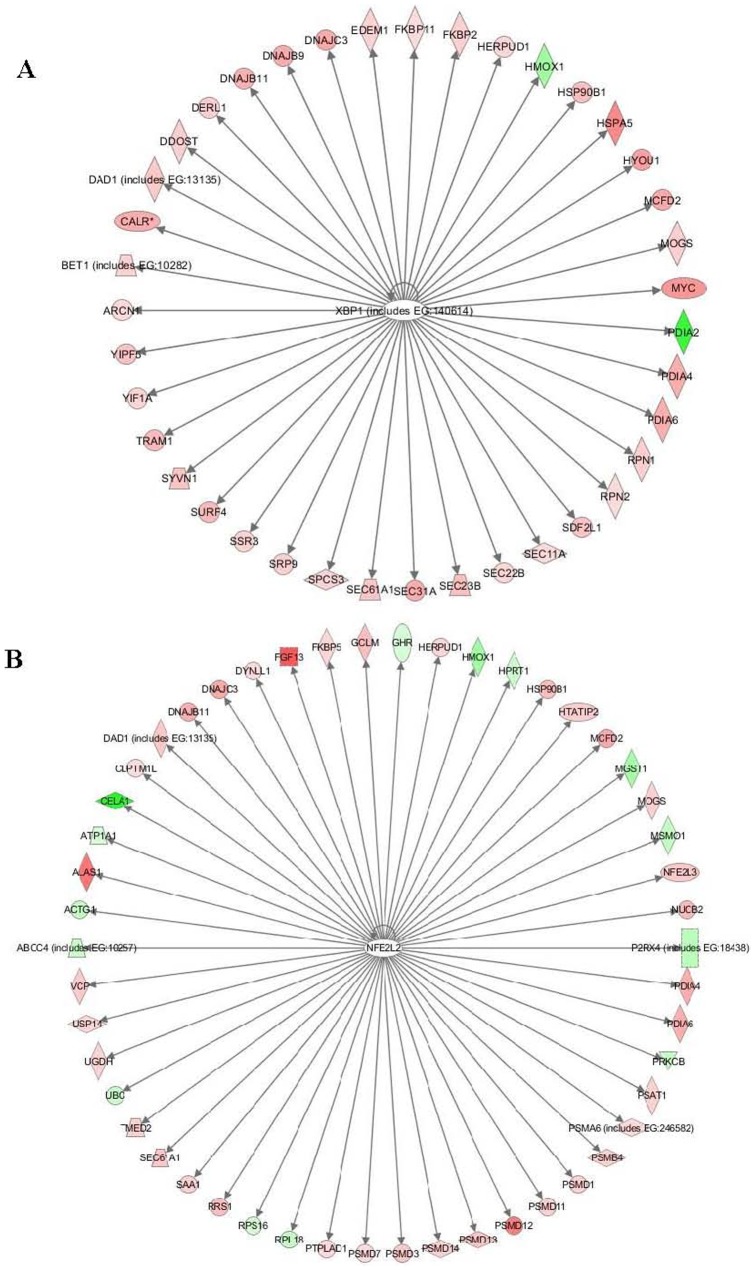
Two most significant transcription factor networks in the zebrafish liver in response to TCDD exposure. (A) Xbp1 network. (B) Nfe2l2 network, Target genes in the differentially expressed gene list (Table S1) are shown with red color indicating up-regulation and green color down-regulation. The intensity of the color corresponds to the relatively levels of up- and down-regulation.

By IPA analysis, six significant transcription factors were found to be related to TCDD-induced hepatotoxicity (Table 4), including Xbp1, Nfe2l2, Nr5a2, Ptf1a, Tp53 and Mycn. In particular, the two up-regulated Xbp1 and Nfe2l2 networks were highly enriched with P-values of 1.47E-16 and 4.73E-11 respectively, In the Xbp1 network, 38 target genes were found from the differentially expressed gene list and almost all of them (36) were up-regulated (Figure 3A), indicating that Xbp1 plays a central role in regulating a battery of genes responsible for protein trafficking and secretion [29]. In the Nfe2l2 network, 36 up- and 14 down-regulated genes were induced in the differentially expressed gene list (Figure 3B). This is in consistence with a previous study [30] that most of the regulated genes (such as *mgst1, usp14, herpud1, dnajc3, actg1, hmox1, dnajb11,* etc.) by Nfe2l2 are related to oxidative stress and mediate transcriptional events that facilitate protective responses in animal models exposed to xenobiotic.

**Table 4:** Most significant transcription factors in zebrafish liver after TCDD treatment.

GI_ID	Gene Symbol	Gene Name	Regulation Z-Score	P-value of Overlap
18859572	xbp1	X-box binding protein 1	4.07	1.47E-16
33504556	nfe2l2	nuclear factor (erythroid-derived 2)-like 2	2.45	4.73E-11
24158438	nr5a2	nuclear receptor subfamily 5, group A, member 2	−2.59	1.05E-03
124249093	ptf1a	pancreas specific transcription factor, 1a	−2.79	7.64E-06
18859502	tp53	tumor protein p53	−3.035	2.73E-02
47271377	mycn	v-myc myelocytomatosis viral related oncogene, neuroblastoma derived (avian)	−3.55	4.47E-04

### Deregulated pathways in zebrafish livers treated by TCDD

To investigate the change of molecular pathways induced by TCDD treatment, GSEA was performed using the set of differentially expressed genes (651 up and 407 down) and 163 up- and 123 down-regulated pathways were identified (Table S3). Among all pathways, five up- and eight down-regulated pathways had significant FDR values less than 0.25 and are shown in Table 4. The first three pathways are associated with cell cycle progression, which have also been reported in mammalian models under TCDD stress [31], [32]. However in the present study, cell cycle and related pathways were significantly enhanced while some mammalian studies showed a decrease of these activities, suggesting different regulatory mechanism in various models as well as by different treatment regimes.

The mechanisms responsible for TCDD toxicity are always associated with its ability to disrupt endocrine functions. In our data, ubiquitin-proteasome pathway was significantly up-regulated in fish liver after TCDD treatment. Consistent with another study in the mammalian system [33], the up-regulated proteasome and related pathways in our study further indicated that the TCDD-induced ubiquitin-proteasomal degradation of AhR influenced the nucleus transcription by controlling the level of ligand-activated AhR. As ubiquitin-proteasome pathway is significantly induced in zebrafish and all mammalian models analyzed in our study, it is an apparently conserved mechanism of TCDD-induced toxicity between fish and mammals. Prostaglandin synthesis regulation pathway was suppressed in TCDD-treated zebrafish, consistent with a previous report that prostanoid synthesis pathway could be regulated by COX2-TBXS-TP (cyclooxygenase2-thromboxane A synthase1-thromboxane receptor) in zebrafish after TCDD treatment [34].

In the present study, several pathways related to signal transduction were significantly inhibited in zebrafish after TCDD treatment, including Ca^2+^ regulation pathways, Fas signaling pathway, T-cell signal transduction and T-cell receptor signaling pathway, ribosomal protein and its related pathways. TCDD has been reported to significantly increase intracellular free Ca^2+^ in many cell culture systems [35] and to trigger Ca^2+^-mediated endonuclease activity leading to apoptosis [36]. Our study, consistent with several previous reports [37]–[40], indicated that Ca^2+^ regulation pathways could be commonly involved in TCDD action. Furthermore, Fas signaling pathway, T-cell signal transduction and T-cell receptor signaling pathway were also suppressed in our study. Fas, a member of the tumor necrosis factor receptor (TNFR) family, contains a death domain which is essential for the delivery of the death signal [41]. Thus, Fas signaling regulation could be a mechanism of impaired T-cell related pathway induced by TCDD, which are consistent with another previous report [41]. Moreover, ribosomal proteins, in conjunction with rRNA, are involved in the cellular process of translation [42]. Interestingly, the ribosomal protein and its related pathway were down-regulated in zebrafish liver after TCDD exposure, but were induced in other mammalian models [43].

Furthermore, three immune-related pathways were significantly repressed in the zebrafish liver after TCDD exposure (Table 4), including natural killer cell mediated cytotoxicity, T-cell signal transduction and T-cell receptor signaling pathway. Moreover, several B-cell related pathways were also found to be inhibited with FDR>0.25 (Table S3). Collectively, our data indicate that hepatic immune-related functions were impaired in fish exposed to TCDD.

### Toxicogenomic comparison between zebrafish and mammalian models

**Figure 4:**
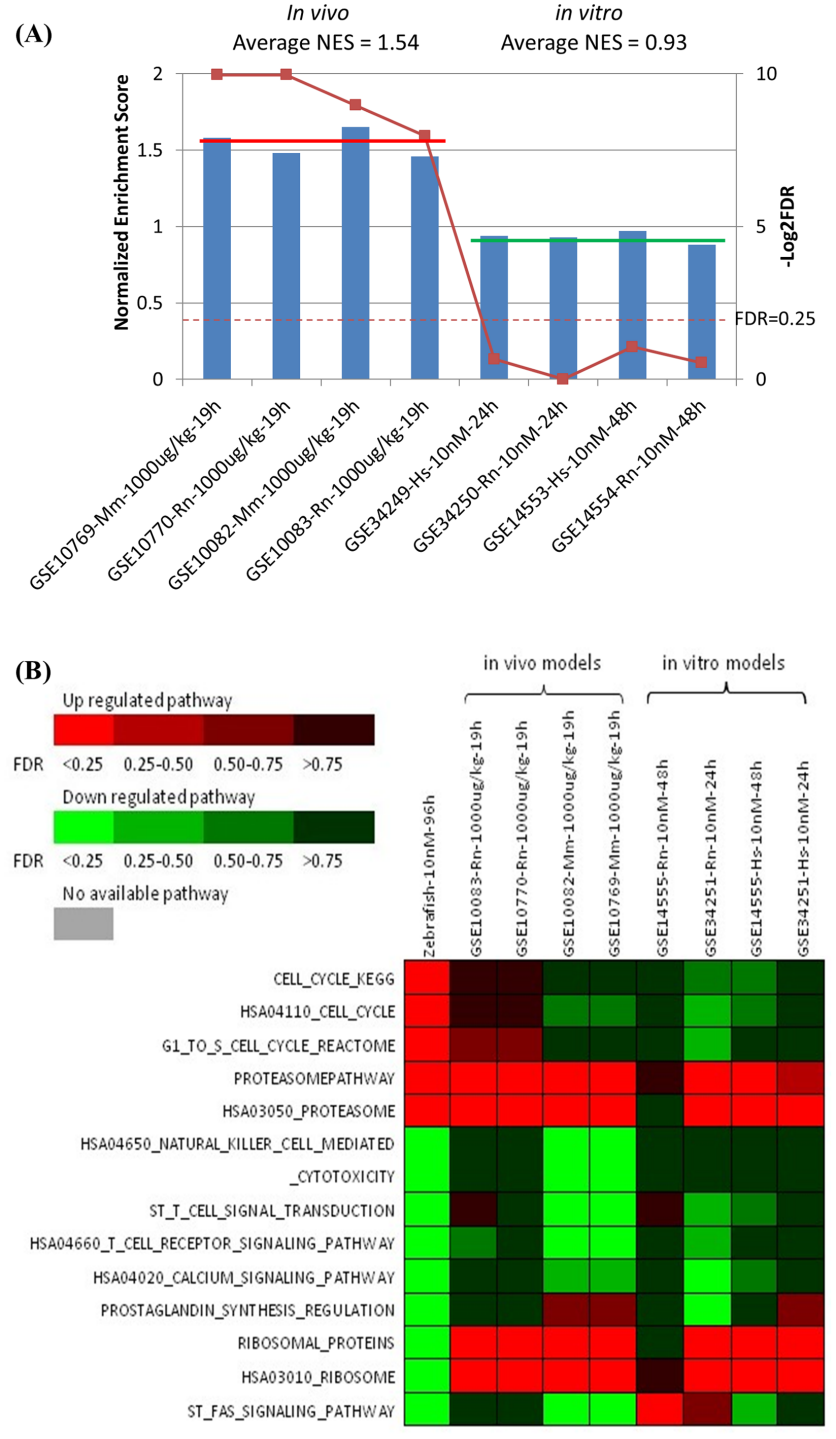
Comparative analyses of zebrafish and mammalian transcriptomic data from TCDD treatments. (A) Correlation of hepatic transcriptome changes in the zebrafish with the mammalian in vivo and in vitro models by GSEA analysis. –Log2FDR = 2, that means FDR = 0.25. (B) Comparison of the pathways in zebrafish and the mammalian models by GSEA analysis. The heat map includes the significant pathways in zebrafish and mammalian models treated by TCDD, the criteria of zebrafish pathways is FDR<0.25.

In order to gain insight into the common molecular toxicity of TCDD between fish and mammals as well as the validity of the zebrafish model to predict chemical toxicity for risk assessment for human health, our transcriptomic data were compared with available transcriptomic data from both in vivo and in vitro mammalian studies with TCDD. Among 26 related series in the GEO database using human cell lines, rat and mouse tissues and cells based on microarray studies, we found six series (GSE10082, GSE10083, GSE10769, GSE10770, GSE14555 and GSE34251) with effective comparability (Table 5) while other series were not included in the comparative analysis due to incompatibility in data uploading, platform, experimental strategy, etc. The list of 650 up-regulated genes from the current zebrafish study was used to represent the zebrafish transcriptome in GSEA. As shown in Figure 4A, based on normalized enrichment scores (NES) and false discovery rate (FDR), the zebrafish hepatic genes expression showed more resemblance to the in vivo models (average NES is 1.54) than in vitro models (average NES is 0.93). The comparison with all of the four in vivo data, FDR was smaller than 0.25, while none of the in vitro data showed such significance. These observations indicate that the zebrafish data is more similar to the in vivo mammalian data than in vitro data, further enforcing the validity of the zebrafish model as a potentially high-throughput and economic in vivo experimental models for studies relevant to human health.

**Table 5:** Detail information of GSE series from GEO database used in this study.

GSE series no.	Animal models	Treated concentrations	Treated time	Strategy
GSE10083	Rat, *Rattus norvegicus* (liver)	1000ug/kg	19h	in vivo
GSE10770	Rat, *Rattus norvegicus* (liver)	1000ug/kg	19h	in vivo
GSE10082	Mouse, *Mus musculus* (liver)	1000ug/kg	19h	in vivo
GSE10769	Mouse, *Mus musculus* (liver)	1000ug/kg	19h	in vivo
GSE14555	Rat, *Rattus norvegicus* (Primary Rat Hepatocytes)	10nM	48h	in vitro
GSE34251	Rat, *Rattus norvegicus* (Primary Rat Hepatocytes)	10nM	24h	in vitro
GSE14555	Human, *Homo sapiens* (Primary Human Hepatocytes)	10nM	48h	in vitro
GSE34251	Human, *Homo sapiens* (Primary Human Hepatocytes)	10nM	24h	in vitro

To further analyze the correlation of zebrafish and other mammalian models after TCDD treatment, comparison of pathways were made by GSEA pre-ranked function. One up-regulated (Proteasome pathway) and three down-regulated pathways (HSA04650 Natural killer cell mediated cytotoxicity, HSA04660 T cell receptor signaling pathway, HSA04020 Calcium signaling pathway) in zebrafish showed the same regulation direction as those in all other mammalian models, indicating that the four pathways were well conserved in vertebrates. Meanwhile, HSA03010 Ribosome pathway displayed completely opposite regulated trends between zebrafish and all mammalian models we compared (Figure 4B), indicating the species-specific patterns between fish and mammals after TCDD treatment, which need further analysis in future. Moreover, after comparing the conserved pathways (the same regulation direction pathways) among zebrafish model, *in vivo* and *in vitro* mammalian models, we noted that the degree of regulation is more consistent between the zebrafish and in vivo mammalian models than between the zebrafish and in vitro mammalian models, as indicated by the color codes in Figure 4B.

In summary, by RNA-sequencing based transcriptomic analyses, we have carried out detailed analyses of TCDD-induced molecular changes in zebrafish. Other than the well characterized AhR pathway and *cyp1a1* biomarker gene induced by TCDD, we also found some new biomarker genes that have been validated from independent experiments. Interestingly, some of the new biomarker genes encode secreted proteins such as Fgf13b, Sdf2 and RNasel2, which may by analyzed from serum samples by a non-invasive approach. Further and comparative transcriptomic analyses of our zebrafish data and available mammalian data from TCDD treatment experiments indicate several well conserved TCDD-responsive pathways, including up-regulated proteomic pathway and several down regulated pathways such as calcium signaling pathway, natural killer cell mediated cytotoxicity, T-cell receptor signaling pathway etc. Furthermore, GSEA analyses indicate that the TCDD-induced zebrafish transcriptomic data is more similar to in vivo mammalian data than in vitro data, thus indicating the validity of the zebrafish model as a valuable in vivo model to infer molecular toxicity relevant to human health.

## Supporting Information

Table S1 **Complete list of differentially expressed genes in zebrafish liver exposed to TCDD with a cut off of fold change>2 and TPM >10.**
(XLSX)

Table S2 **Up-regulated CYP family genes in zebrafish treated by TCDD.**
(XLSX)

Table S3 **Complete list of pathways in zebrafish exposed to TCDD as revealed by GSEA analysis.**
(XLSX)
